# Biphasic Cuirass Ventilation for Airway Surgeries: A Comprehensive Review

**DOI:** 10.7759/cureus.75477

**Published:** 2024-12-10

**Authors:** Abhay Bodhey, Manjusha Bodhey, Nabil A Shallik, Mansour Al Nadhari, Hany F Griess, Osama Al Ani, Shaji Sainuddin, Kurian P Thomas, Osama ElHanfi, Suraj Jose, Abhijit Nair

**Affiliations:** 1 Anesthesiology, Rashid Hospital and Trauma Centre, Mohammed Bin Rashid University of Medicine and Health Sciences, Dubai Health, Dubai, ARE; 2 Anesthesiology, Dubai Hospital, Dubai, ARE; 3 Anesthesiology, Hamad Medical Corporation, Doha, QAT; 4 Anesthesiology, Weill Cornell Medical College, Doha, QAT; 5 Anesthesiology, Qatar University, Doha, QAT; 6 Anesthesiology, Tanta University, Tanta, EGY; 7 Anesthesiology, Ibra Hospital, Ibra, OMN

**Keywords:** airway access, airway surgeries, critical airway, ent anesthesia, ent surgery, negative pressure ventilation

## Abstract

Airway surgeries pose great challenges for the anesthesiologists as the airway is shared by them and the surgeon. It is of paramount importance to have control of the airway during such surgeries. Many techniques have been employed to provide uninterrupted oxygenation to the patient with or without the presence of a definitive airway. Recently, biphasic cuirass ventilation (BCV) has been used effectively to provide tubeless airway management in patients undergoing airway surgeries. This article discusses the feasibility and viability of using BCV for various airway surgeries and also reviews the existing literature regarding its use in the perioperative period for such indications.

## Introduction and background

Airway management and ventilation are challenging in various ear, nose, and throat (ENT) surgeries, especially airway surgeries as the airway is shared with the operating surgeon too. Maintaining adequate depth of anesthesia as well as preserving spontaneous ventilation is not only difficult but may also cause respiratory or cardiac depression. Both teams must have access to the airway during laryngeal, tracheal, or pharyngeal surgeries for optimal oxygenation and ventilation. For the surgeon to operate, the anesthesiologist frequently has to remove the endotracheal tube, which increases the possibility of hypoxia or loss of airway control. Alternative methods, like apneic oxygenation, high-frequency jet ventilation (HFJV), and spontaneous respiration using intravenous anesthesia and high-flow nasal oxygen (STRIVE-Hi) technique, may be utilized when traditional ventilation is not feasible [1]. These techniques can enable surgery to proceed without interruption, but they are also associated with adverse events owing to hypercarbia and barotrauma and thus need careful observation.

Careful planning, closed monitoring, anticipation of serious events, and emergency management readiness are key to ensuring patient safety in these complex cases. Jet ventilator or Sander’s injector can also be used through the bronchoscopes, but complications such as barotrauma, pneumothorax, or mediastinal emphysema can occur because of high airway pressures [2,3]. Although a micro-laryngeal endotracheal tube improves the surgical field in laser surgery, it can also be a fire hazard [4]. Tritube is an ultrathin ventilation tube with an outer diameter of only 4.4 mm and an internal diameter of 2.4 mm. Tritube can be used exclusively with Evone or Ventrain ventilator [5].

During airway surgeries, biphasic cuirass ventilation (BCV) has become a viable alternative that provides efficient ventilation without requiring access to the airway [6]. BCV is a negative pressure ventilation system using an extrathoracic ventilation device applied to such patients [7,8]. In this review article, we reviewed the existing literature on the safety and efficacy of using BCV in airway surgeries.

## Review

Negative-pressure ventilation

The first portable negative/positive pressure cuirass ventilator, which was patented in 1901 by a Hungarian doctor named Rudolph Eisenmenger, was used for cardiopulmonary arrest due to drowning or intoxication [9]. It was a two-part box that enclosed the chest and abdomen while leaving the throat and limbs free. The resuscitation of a man who had hung himself was described as extremely successful at that time. In 1904, a foot-operated bellow was replaced by motors called the Biomotor. Drinkers' respirator, popularly called the 'Iron Lung,' was responsible for providing respiratory support during the polio era experienced during the 1930s. Modern critical care and anesthesia workstation settings mostly use positive-pressure ventilation (PPV). In contrast to PPV, several studies have shown that NPV has a smoother pressure-volume curve, more homogenous inflation, less local and bulk strain concentration, less hysteresis, less energy dissipation, and less tissue relaxation [6-9]. The concept behind NPV is that an intermittent negative pressure generated outside the thorax triggers inspiration, which causes the lungs to expand and draw in air [10]. The expiratory phase of conventional NPV depends on the passive recoil of the chest wall, which restricts the ventilation frequency to about 30 breaths per minute. Using a negative pressure during the inspiratory phase and a positive pressure to compress the abdomen during expiration, modern negative pressure ventilators can regulate both inspiration and expiration without depending on the chest's passive recoil. Consequently, high ventilation frequencies are possible, and both the expiratory and inspiratory phases are completely controlled [11].

BCV for Airway Surgeries

Sharing the airway during ENT and airway surgery with the surgeons is a big concern for anesthesiologists. This concern can be addressed only by good communication between the two teams [12]. BCV provides a useful substitute for conventional ventilation techniques in ENT and airway surgeries. BCV not only keeps the airway free from any instrumentation with airway adjuncts like an endotracheal tube but also allows an uninterrupted surgical field, thereby reducing conflicts. Its capacity to deliver non-invasive ventilation while preserving an open surgical field improves surgical results, lowers postoperative complications, and increases patient safety. However, cautious patient selection, specific training, and meticulous teamwork are necessary for the effective use of BCV in these surgical settings. BCV technology is expected to become ever more important in the perioperative management of complex airway surgeries as it develops further, opening up new avenues for enhancing surgical results and patient care.

The Modality of BCV

BCV functions by promoting the lungs' regular activity. Natural breathing leads to the chest wall expansion causing flattening of the diaphragm due to the contraction of respiratory muscles. Passive exhalation occurs due to relaxation of the respiratory muscles. As a noninvasive method of ventilation, BCV reproduces this process and helps individuals who require respiratory support for the short or long term. BCV can be used to facilitate high-frequency chest wall oscillation, synchronized ventilation, controlled ventilation, negative pressure ventilation, and coughing to clear sputum [13,14].

The Equipment

**Figure 1:**
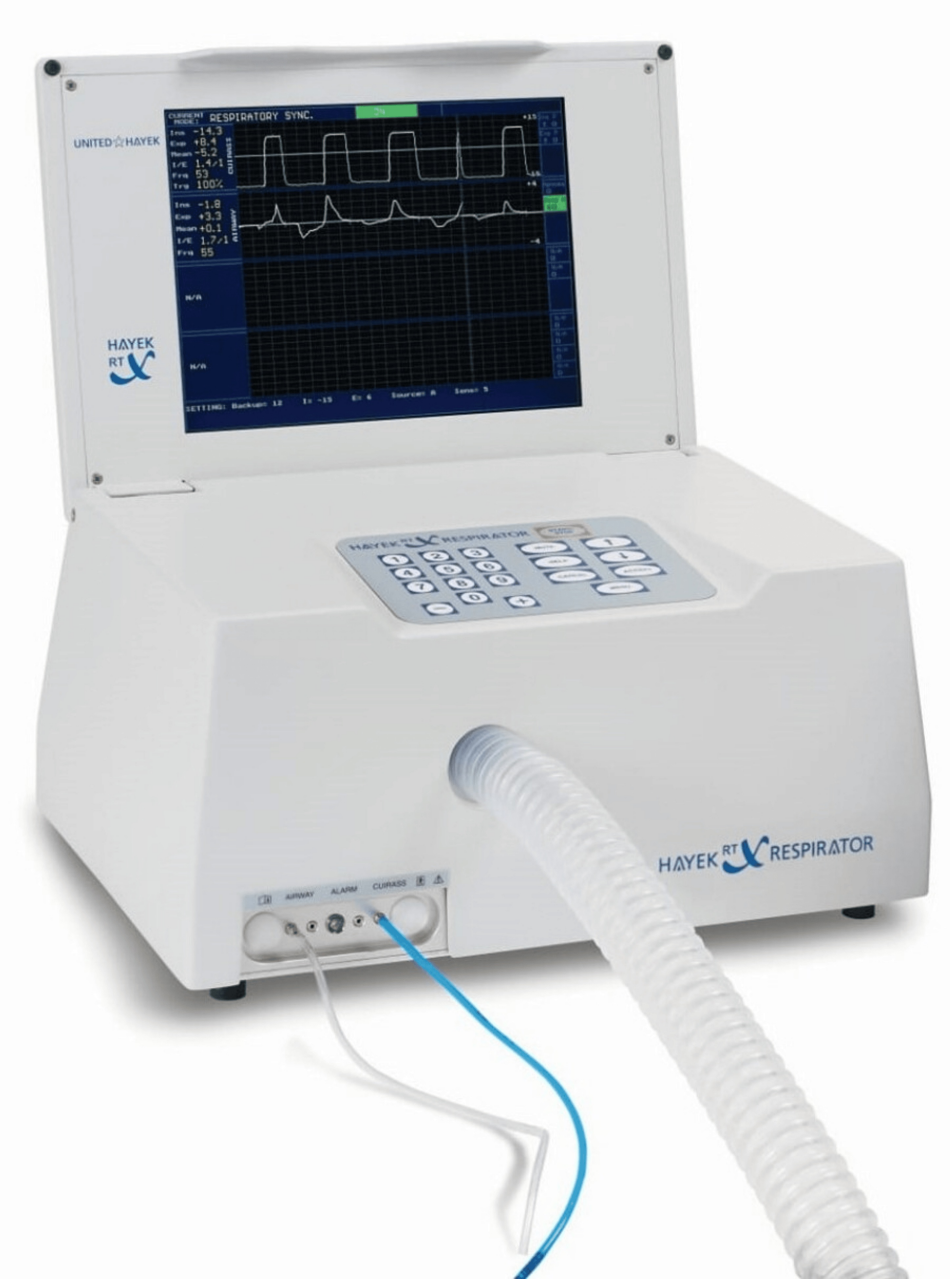
Picture showing Hayek respirator used for BCV. BCV: biphasic cuirass ventilation (Image reproduced with permission from United Hayek Industries)[17].

**Figure 2:**
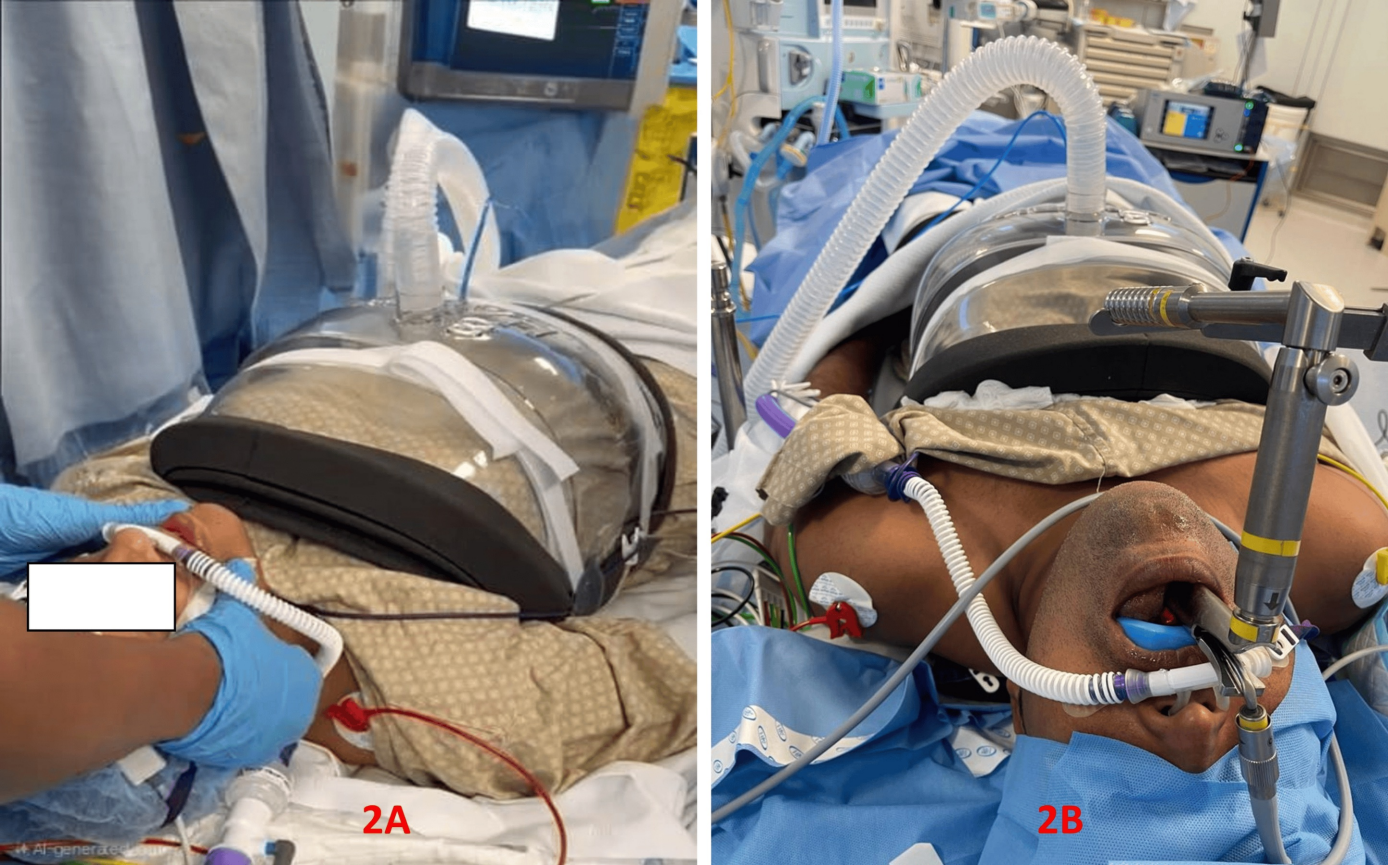
Airway surgery with BCV. A: patient with BCV attached for surgery with HFNO for oxygen supplementation, B: patient undergoing airway surgery. BCV: biphasic cuirass ventilation; HFNO: high flow nasal oxygenation (Images are from the authors' own database).

**Figure 3:**
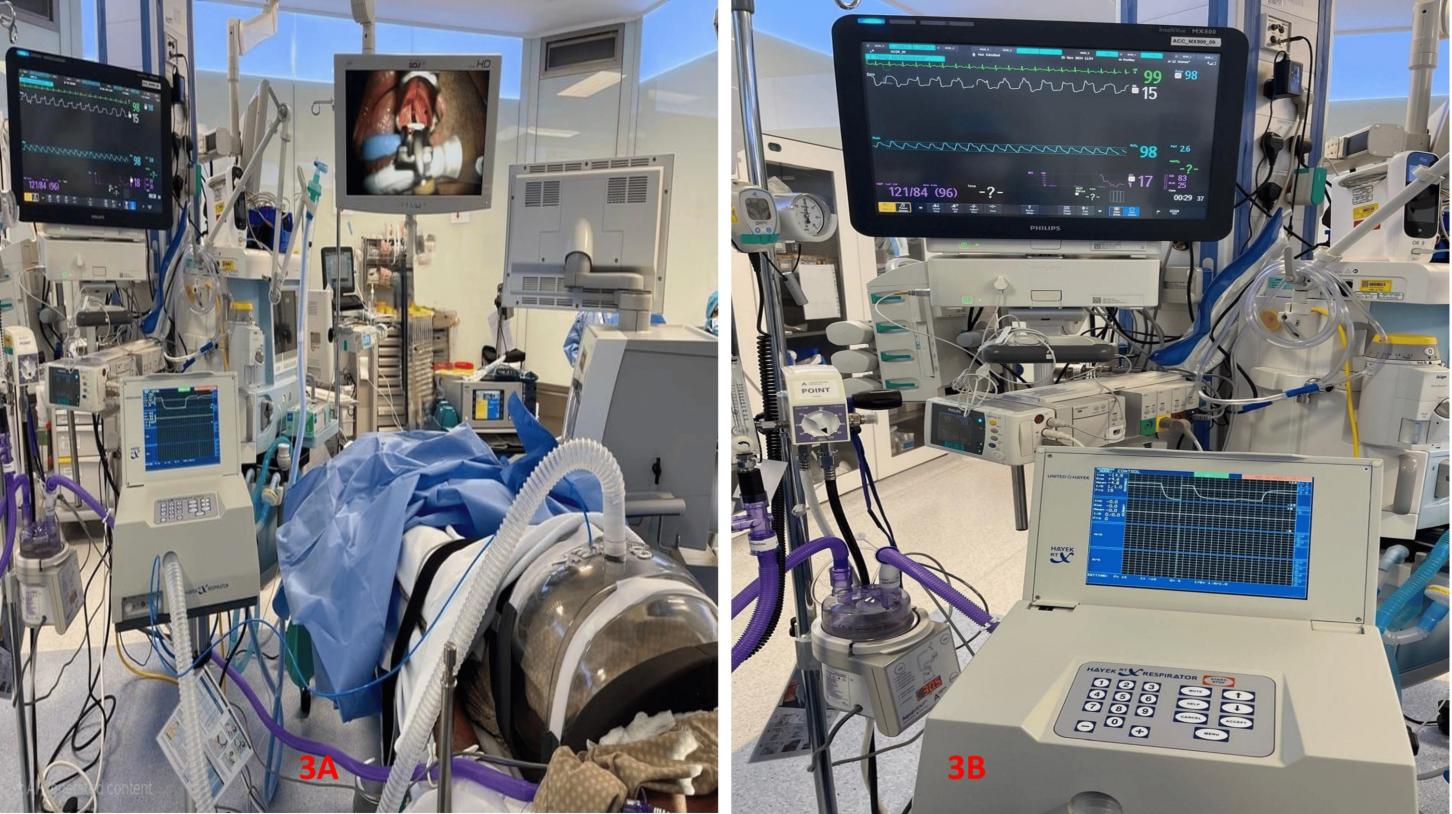
Intraoperative images during airway surgeries. A: Image showing operation theatre during airway surgery with BCV in use, B: Image showing BCV in use and intraoperative monitoring. (Images are from the authors' own database).

A shell-like device called a cuirass is placed over the chest and upper abdomen as part of the BCV ventilation technique. The apparatus simulates the diaphragm's natural movement during respiration by producing a cycle of positive and negative pressure (Figure 1). While the positive pressure makes expiration easier, the negative pressure causes inspiration by expanding the chest cavity. It has been demonstrated that more physiological breathing is possible with this biphasic mechanism than with conventional positive pressure ventilation (PPV) [15,16]. When BCV is used for airway surgeries (vocal cord polyp excisions or vocal cord augmentation) as a mode of ventilation, the need for tracheal intubation or other invasive airway maintenance techniques is eliminated. This is very helpful in various airway surgeries where the surgical team needs to have unhindered access to the airway (Figures 2A, 2B and Figures 3A, 3B).

The Hayek RTX series of ventilators was invented by the late Dr. Zamir Hayek, a world-renowned pediatrician and respiratory care specialist who was also a pioneer in the field of cuirass ventilation [17]. The initial biphasic systems were bulky and not very portable. However, clinicians managed to use them effectively in the successful anesthesia management of airway surgeries for various indications.

The machines that provide BCV have greatly improved in terms of portability and have many advanced features, making it easier to use for a variety of acute and chronic conditions, including airway surgeries. The current BCV machines are fully computerized, relatively easy to set up, and easy to operate. The available modes include ventilatory synchronized, secretion clearance, continuous negative, and mandatory controlled ventilation. There are currently seven pediatric and four adult-sized cuirasses available for BCV.

Summary of published studies

Dilkes et al. recruited 25 patients (nine women and 16 men), all of whom needed an upper airway endoscopy [8]. The patients were between 25 and 75 years old and weighed between 52 and 105 kg. For all of these patients, BCV was used. A baseline end-tidal carbon dioxide (ETCO_2_) was taken after anesthesia was induced with suxamethonium and propofol, and a nasal airway was inserted. Endoscopy was performed for various vocal cord pathologies such as polyps, nodules, edema, suspected malignancy, and hemangiomas. Based on the findings of a nasal endoscopy showing a large mass at the epiglottis that obstructed the airway, Broomhead et al. reported using the Hayek oscillator in a patient with a difficult airway [6]. Based on the morphology and location of the growth, securing an airway was even impossible with a fiberoptic bronchoscope. Propofol and atracurium were used to induce general anesthesia, and the lungs were manually ventilated until the Hayek oscillator was started. The procedure to debulk the tumor took forty minutes and went uneventfully. Monks et al. utilized a Hayek oscillator without using an endotracheal tube in 41 patients undergoing micro-laryngeal surgery [7]. The initial parameters consisted of an I:E ratio of 1:1, an inspiratory pressure of -24 cm of water, an expiratory pressure of +10 cm of water, a rate of 90 cycles per minute, and a change in the respiratory rate as required. Mask ventilation was used to control the airway until the surgeons introduced the scope for the micro-laryngeal procedure.

Kristensen et al. published their experience with 14 BCV-assisted airway surgeries [18]. When it was not possible to secure the airway with a definitive airway, they chose the cases based on the difficulty anticipated with a definitive airway management. The patients also had a history of airway surgeries and had faced repeated intraoperative extubation and intubation, which resulted in morbidity. An additional criterion was when an extended surgical procedure was anticipated. The authors managed all the cases successfully without any adverse event, either airway-related or otherwise. In a series of three patients having tracheobronchial stenting, Mori et al. used BCV as a modality for airway management [19]. When positive pressure ventilation was not an option during tracheobronchial stenting procedures, BCV helped with spontaneous breathing. The authors did not suffer from severe respiratory acidosis or hypoxemia in any of the three cases (Table 1).

**Table 1:** Summary of published articles in which BCV was used for airway surgeries. BCV: biphasic cuirass ventilation.

Authors	Year	Country	Number of patients	Indications	Summary
Dilkes et al [8]	1993	UK	25	Microlaryngeal surgeries for vocal cord pathologies like polyps, nodules, edema, suspected malignancy, and hemangiomas	It is a safe method of ventilation for airway surgeries, with the advantage of dispensing with any form of endolaryngeal or endotracheal intubation
Broomhead et al [6]	1995	UK	1	Pharyngeal tumor debulking	Securing the airway with a fiberoptic bronchoscope was unsuccessful. Eventually, the surgery was performed without a tube, using BCV.
Monks et al [7]	1995	UK	41	Laryngeal surgeries	Provided excellent gas exchange and stable hemodynamics without major adverse events.
Kristensen et al [18]	2023	Denmark	14	Laser evaporation/shaving of lesions, tracheal stenosis, steroid injection, balloon dilatation	Provided good conditions for peri-operative anesthesia management and uninterrupted surgical access.
Mori et al [19]	2017	Japan	3	Tracheobronchial stent insertion or removal by a rigid bronchoscope	BCV was useful for maintaining adequate ventilation during anesthesia for the airway procedures, and no there were no significant complications.

Perioperative management of patients undergoing airway surgeries with BCV

Preoperative Evaluation

A comprehensive evaluation is essential because airway surgeries frequently involve a compromised or challenging airway. The anatomy of the airways and any obstructions can be evaluated using any form of imaging that is currently available to the patient, such as computed tomography (CT) or magnetic resonance imaging (MRI). It is essential to determine whether the patient has any co-existing medical conditions that could impact their ventilatory needs, such as obesity, asthma, or chronic obstructive pulmonary disease (COPD). Laryngeal surgeries, like microlaryngeal surgery, laser surgery, and vocal cord procedures, require precise airway management. BCV provides continuous ventilation without an endotracheal tube, providing surgeons with a clear, uninterrupted, and unobstructed view of the larynx. Maintaining an open airway is critical in surgeries involving the trachea or subglottic region. BCV enables effective ventilation without tracheal intubation. BCV provides improved patient safety, better surgical outcomes, and reduced postoperative complications in the indications mentioned above. 

Anesthesia Management

Following the connection of necessary monitoring devices such as a pulse oximeter, electrocardiogram, non-invasive blood pressure monitoring cuff, and transcutaneous CO_2_ if available, an opioid such as fentanyl/sufentanil, and an induction agent such as propofol. The neuromuscular blockade (suxamethonium, atracurium, cisatracurium, or rocuronium) could be used at the discretion of the anesthesiologist or based on the requirements. High-flow nasal oxygenation (HFNO) or a facemask is used for oxygen insufflation, and the BCV is started simultaneously. Propofol target-controlled infusion (TCI) with bispectral index (BIS) monitoring, aimed at a BIS between 50 and 60, is required to maintain general anesthesia. At the attending anesthesiologist's discretion, boluses of fentanyl or a TCI of remifentanil and/or dexmedetomidine may also be used. The recovery from anesthesia is crucial and needs to be done meticulously, avoiding any respiratory events like stridor or desaturation. The neuromuscular blockade needs to be reversed completely if administered. BCV can be continued in the postoperative period in case of prolonged surgery, in the presence of any respiratory comorbidities, or when any untoward events are anticipated. The anesthesiologist and the surgical team must work as a team during airway surgeries utilizing BCV. Although BCV's non-invasive nature provides a major benefit in these procedures, patient safety depends on meticulous planning, monitoring, and preparedness for airway emergencies.

Scope of BCV in Routine Practice

Not all patients undergoing ENT or airway surgeries are suitable candidates for BCV. Careful patient selection is essential to ensure the safety and efficacy of this ventilation method. Patients with severe chest wall deformities, open wounds, presence of hiatus hernia, pregnant ladies, not fasting, or those who require high levels of positive end-expiratory pressure (PEEP) are not ideal candidates for BCV. BCV use necessitates particular equipment and education. To guarantee a smooth integration with the surgical procedure, the surgical team must work closely with anesthesia providers who are knowledgeable about the operation and monitoring of BCV systems. Another factor that may prevent BCV equipment from being widely used is its limited availability in all healthcare settings. Careful preparation and planning are needed to incorporate BCV into current perioperative practice. The surgical team must understand the unique needs and constraints of BCV, and anesthesia teams must be ready to make the shift to traditional ventilation techniques if needed. This degree of coordination is necessary to guarantee the safe and efficient use of BCV during surgery.

Limitations

This review has certain limitations. We have reviewed only case series as there are no randomized controlled trials published to date. Hence, a control group was lacking. Therefore, the safety and efficacy of BCV for all airway surgeries cannot be established with the present review. Moreover, the overall sample size in terms of the number of cases in which BCV was used is very small. 

There are certain restrictions when using BCV in conjunction with airway surgery. As there is no tube, the airway is unprotected, thus making it prone to aspiration, either with blood/secretions or tissue. The onus lies with the surgeon to prevent anything from entering the unprotected airway. Continuous monitoring of ETCO_2_, which is a standard of care in anesthesia practice, is not possible, and therefore, there is an emphasis on having a transcutaneous carbon dioxide (CO_2_) monitoring facility. Since transcutaneous CO_2_ measurement or repeated arterial blood gas samples are required, it is not feasible to measure the ventilatory minute volume directly during surgery. A face mask or supraglottic airway device can be used to measure the minute volume of cuirass ventilation as a baseline before inserting the suspension laryngoscope. Because the cuirass is positioned above the front surface of the thorax, special preparation is needed when placing the suspension laryngoscope. To date, there has been no investigation into the role of cuirass ventilation in patients undergoing airway surgery who are morbidly obese and also in pediatric patients. Thus, BCV is probably not appropriate for every patient undergoing airway surgery. Moreover, as the use of BCV needs specialized equipment, the attending anesthesiologist and the anesthesia nurse/technician should be appropriately trained in using it perioperatively. 

## Conclusions

BCV appears to be a safe and feasible technique and can be used effectively in patients undergoing complex ENT and airway surgeries if the patient is selected properly. The use of the equipment is easy and can be extended in the perioperative period based on the type of surgery done and the patient's characteristics. Well-designed studies are necessary to compare BCV with other modes of airway management for airway surgeries to establish its safety and efficacy.
